# Addition of Plasma Myeloperoxidase and Trimethylamine N-Oxide to the GRACE Score Improves Prediction of Near-Term Major Adverse Cardiovascular Events in Patients With ST-Segment Elevation Myocardial Infarction

**DOI:** 10.3389/fphar.2021.632075

**Published:** 2021-09-28

**Authors:** Yu Tan, Jinying Zhou, Shujun Yang, Jiannan Li, Hanjun Zhao, Li Song, Hongbing Yan

**Affiliations:** ^1^ Department of Cardiology, Fuwai Hospital, National Center for Cardiovascular Diseases, Peking Union Medical College and Chinese Academy of Medical Sciences, Beijing, China; ^2^ Department of Cardiology, Xiamen Cardiovascular Hospital, Xiamen University, Xiamen, China; ^3^ Department of Cardiology, Fuwai Hospital, Chinese Academy of Medical Sciences, Shenzhen, China

**Keywords:** myeloperoxidas, trimethylamine N-oxide, acutemyocardial infarction, risk predication, biomarker, grace

## Abstract

**Background:** The Global Registry of Acute Coronary Events (GRACE) risk score (GRS) is an established powerful model in predicting prognosis of patients with acute coronary syndrome. However, it does not contain pathophysiological biomarkers. Myeloperoxidase (MPO) and trimethylamine N-oxide (TMAO) are novel biomarkers of different pathophysiological processes of acute myocardial infarction, and each of them predicts risk of adverse clinical outcomes. We aimed to investigate whether the addition of MPO and TMAO could improve a GRS-based prediction model in patients with ST-segment elevation myocardial infarction (STEMI).

**Methods:** A prospective cohort of 444 consecutive patients with STEMI who underwent primary percutaneous coronary intervention were enrolled in this study. Plasma levels of MPO and TMAO were measured using samples collected before the interventional procedure. GRS at admission was calculated. Death and nonfatal myocardial infarction were recorded as major adverse cardiac events (MACEs). Kaplan–Meier survival analysis with Cox proportional-hazards regression was used to identify predictive values of MPO and TMAO. Area under the receiver-operator characteristic curve (AUC) and net reclassification improvement (NRI) were calculated to evaluate the increment of predictive value for the combination of MPO and TMAO with GRS in predicting adverse clinical outcomes.

**Results:** During 6 months follow-up, 27 patients suffered MACEs. Both MPO (hazard ratio [HR]: 2.55, 95% confidence interval [CI]: 1.11–5.87; *p* < 0.05) and TMAO (HR: 4.50, 95% CI: 1.78–11.40, *p* < 0.01) predicted MACEs at 6 months. The AUC for MPO, TMAO, GRS, and their combination in predicting risk of MACEs at 6 months is 0.642, 0.692, 0.736, and 0.760, respectively. The addition of MPO and TMAO significantly improved the net reclassification of GRS for predicting MACEs at 6 months (NRI: 0.42, *p* = 0.032).

**Conclusion:** Plasma MPO and TMAO each predict near-term risk of adverse outcomes in patients with STEMI. Furthermore, the combination of MPO and TMAO with GRS enables more accurate prediction of cardiovascular events compared with GRS alone.

## Introduction

The Global Registry of Acute Coronary Events (GRACE) risk score (GRS) is a powerful model in predicting short- and long-term mortality and reinfarction after acute coronary syndrome (ACS). However, it does not contain biomarkers that reflect diverse pathophysiological processes of ACS patients.

Myeloperoxidase (MPO) and trimethylamine N-oxide (TMAO) are novel biomarkers of different pathophysiological processes of acute myocardial infarction (AMI). Circulating MPO level is elevated in patients with plaque erosion compared with those with plaque rupture. (Ferrante et al., 2010; Tan et al., 2020a). The systemic level of TMAO is higher in patients with plaque rupture than in those with plaque erosion. (Fu et al., 2016; Tan et al., 2019; Tan et al., 2020b). Moreover, each of them independently predicts the risk of adverse cardiovascular events in patients with ACS (Baldus et al., 2003; Brennan et al., 2003; Khan et al., 2007; Mocatta et al., 2007; Li et al., 2017; Suzuki et al., 2017). However, the utility of these biomarkers in combination is unknown.

In this study, we investigated the value of MPO, TMAO, and their combination in predicting cardiovascular events in patients with ST-segment elevation myocardial infarction (STEMI). We further explored whether the addition of MPO and TMAO improve prediction ability of GRS for near-term outcomes in STEMI patients.

## Methods

### Study Population

Consecutive patients presenting with STEMI who underwent primary percutaneous coronary intervention at Fuwai Hospital were prospectively enrolled in this study cohort. STEMI was defined as continuous chest pain lasting >30 min, ST-segment elevation >0.1 mV in at least two contiguous leads or new left bundle branch block on 18-lead electrocardiogram, and an elevated troponin I level (cTnI ≥0.08 ng/ml) (Van de Werf et al., 2008). Between March 2018 and March 2019, 444 eligible patients with STEMI were enrolled. This study was performed in accordance with the Declaration of Helsinki and was approved by the Ethics Committee of Fuwai Hospital. All patients provided written informed consent.

### Laboratory Tests

Blood samples were collected *via* radial or femoral access before the interventional procedure using vacutainer tubes containing ethylenediaminetetraacetic acid (EDTA). Samples were maintained at 4°C, processed within 3 h, and then stored at −80°C until further analysis. Plasma levels of TMAO were quantified by stable isotope dilution high-performance liquid chromatography with online electrospray ionization tandem mass spectrometry using an API 3200 triple quadrupole mass spectrometer (AB SCIEX, Framingham, MA) with a d9-(trimethyl)-labeled internal standard as described previously. (Wang et al., 2014a). Plasma MPO was measured by enzyme-linked immunosorbent assay using a commercial kit (DMYE00B, R&D Systems, United States), according to procedures recommended by the manufacturer. Duplicates of 20 randomly selected samples and all samples were measured to evaluate the intra-assay coefficient variation of TMAO and MPO, respectively. The estimated glomerular filtration rate (eGFR; ml/min per 1.73 m^2^) was calculated using the Modification of Diet in Renal Disease study equation (Levey et al., 2009).

### Calculation of GRACE Score

The GRS was calculated at admission on the basis of a web-based GRS calculator for each patient to assess the near-term risk of death or MI. The GRACE risk criteria comprises age, heart rate, systolic blood pressure, initial serum creatinine, Killip class, cardiac arrest on admission, elevated troponin I level, and ST-segment deviation.

### Definition of Endpoints

The primary endpoint was major adverse cardiovascular event (MACE), which included nonfatal MI or death. The secondary endpoint was death. Reviews of medical records and follow-up telephone interviews or clinical visits were conducted for 30-day and 6-month clinical outcomes. A 100% follow-up was achieved.

### Statistical Analysis

Continuous data are presented as mean ± standard deviation or median (interquartile range). Categorical variables are presented as count (percentage). Continuous variables were compared using one-way analysis of variance and the Kruskal–Wallis test for parametric and nonparametric data, respectively. *Post hoc* analyses were performed for continuous variables with a *p* value <0.05 between groups. Category variables were compared by the chi-squared test. Patients were divided into different groups according to median plasma level of MPO and/or TMAO. Kaplan–Meier curves were plotted to estimate the event-free survival between groups using the log-rank test. Cox proportional hazard regression analysis was used to determine hazard ratio (HR) and 95% confidence interval (CI) for adverse outcomes to MPO and TMAO as a continuous variable (log-transformed per SD increase). Adjustments were made for age, sex, smoking, hypertension, diabetes mellitus, eGFR, and N-terminal pro-B-type natriuretic peptide (NT-proBNP). Logistic regression analysis was used to generate predicted probabilities of MPO, TMAO, GRS, and their combination for predicting adverse outcomes. The incremental predictive ability from adding MPO and/or TMAO to GRS was analyzed from those predicted probabilities using increase in the area under the receiver operating characteristic curve (AUC) and category-free net reclassification improvement. (Pencina et al., 2008; Leening et al., 2014). Statistical analyses were performed using SPSS software, version 22 (IBM, Armonk, New York, United States) and SAS software, version 9.4 (SAS Institute Inc., Cary, North Carolina, United States). A two-tailed *p* < 0.05 was considered statistically significant.

## Results

### Baseline Clinical Characteristics

The baseline characteristics of patients stratified according to median plasma levels of MPO (54.3 ng/ml) and TMAO (2.40 μM) are summarized in Table 1. The distribution of MPO and TMAO are shown in Supplementary Figure S1. The intra-assay coefficient variation of MPO and TMAO ranged from 0.9 to 3.3% and 2.2–6.1%, respectively. The mean age of the cohort was 59.8 years, 81.1% were males, 60.1% had hypertension, and 30.2% had diabetes mellitus. Patients with higher plasma levels of MPO and TMAO were more likely to be older, have diabetes mellitus, have reduced renal function, and have higher NT-proBNP level. Eighteen patients had MACEs at 30-day follow-up, including 13 with death, and 27 patients had MACEs at 6-month follow-up, including 18 with death.

**TABLE 1:** Baseline characteristics categorized by median plasma MPO and TMAO levels.

	All patients (*n* = 444)	Group 1 (*n* = 111)	Group 2 (*n* = 221)	Group 3 (*n* = 112)	*p* value*
Age, years	59.8 ± 12.4	58.9 ± 12.2	58.8 ± 12.0^b^	62.3 ± 12.4^c^	0.018
Male, %	81.1	80.2	82.8	78.6	0.623
Medical history, %					
Hypertension	60.1	58.6	62.0	58.6	0.762
Diabetes mellitus	30.2	24.3	31.7	33.3	0.278
Smoking	65.5	63.1	68.3	62.2	0.445
Laboratory tests					
TC, mmol/L	4.4 ± 1.1	4.5 ± 1.2	4.4 ± 1.1	4.2 ± 1.0	0.151
LDL-C, mmol/L	2.8 ± 0.9	2.9 ± 1.0	2.8 ± 0.9	2.7 ± 0.9	0.288
HDL-C, mmol/L	1.1 ± 0.3	1.1 ± 0.3	1.1 ± 0.3	1.0 ± 0.3	0.121
Triglyceride, mmol/L	1.4 (1.0–2.0)	1.3 (1.0–1.8)	1.4 (1.0–2.0)	1.4 (1.0–2.0)	0.554
eGFR, mL/min/1.73 m^2^	93.5 (75.8–108.9)	96.7 (78.5–110.4)	94.7 (78.3–109.1)^b^	85.6 (62.8–101.3)^c^	0.001
NT-proBNP, pg/mL	167.0 (42.8–788.0)	168.3 (34.8–483.0)	121.9 (41.4–743.1)^b^	311.7 (65.1–1376.3)^c^	0.021
MPO, ng/mL	54.3 (34.5–109.7)	35.1 (29.2–43.8)^a^	53.1 (32.1–116.7)^b^	103.4 (73.5–172.5)^c^	<0.001
TMAO, μM	2.40 (1.40–4.05)	1.54 (1.05–1.89)^a^	2.40 (1.27–4.08)^b^	3.97 (3.12–6.58)^c^	<0.001
Discharge medications, %					
Aspirin	97.3	97.3	99.1	93.8	0.018
Statin	96.4	97.3	96.4	95.5	0.779
ACEI/ARB	75.2	76.6	75.6	73.2	0.833
Beta-blocker	87.6	87.4	88.2	86.6	0.910
GRACE score	105 (84–123)	101 (81–120)	103 (85–121)^b^	113 (88–135)^c^	0.002
Endpoints, *n* (%)					
30 days					
MACE	18 (4.1)	0 (0)	7 (3.2)	11 (9.8)	0.001
Death	13 (2.9)	0 (0)	4 (1.8)	9 (8.0)	0.001
6 months					
MACE	27 (6.1)	1 (0.9)	9 (4.1)	17 (15.2)	<0.001
Death	18 (4.1)	1 (0.9)	4 (1.8)	13 (11.6)	<0.001

Continuous data are presented as mean ± standard deviation or median (interquartile range), categorical variables are presented as number (%).TC, total cholesterol; LDL-C, low density lipoprotein cholesterol; HDL-C, high density lipoprotein cholesterol; eGFR, estimated glomerular filtration rate; NT-proBNP, N-terminal pro-B-type natriuretic peptide; TMAO, trimethylamine N-oxide; ACE/ARB, angiotensin-converting enzyme inhibitor/angiotensin receptor blocker; Group 1: both TMAO < median and MPO < median; Group 2: either TMAO > median or MPO > median; Group 3: both TMAO > median and MPO > median.

* Post hoc analyses were performed for continuous variables with a *p* value < 0.05 between groups. Letter a,b, and c represents a *p* <0.05 for group 1 versus 2, group 2 versus 3, and group 1 versus 3, respectively.

### Association Between MPO Level and Clinical Outcomes

Clinical characteristics of the subjects stratified by median plasma MPO levels are shown in Supplementary Table S1. Kaplan–Meier analyses revealed an increased risk for MACEs and death in patients with plasma MPO above median compared with those below median at 30 days (log-rank *p* = 0.017 and log-rank *p* = 0.049, respectively) and 6 months (log-rank *p* = 0.010 and log-rank *p* = 0.016, respectively) (Supplementary Figure S2). Cox regression analyses show that log MPO is a predictor of MACEs and death at 6 months (HR: 2.55, 95% CI: 1.11–5.87, *p* = 0.027; HR: 3.17, 95% CI: 1.18–8.55, *p* = 0.022, respectively) but not at 30 days (HR: 1.72, 95% CI: 0.58–5.09, *p* = 0.331; HR: 2.02, 95% CI: 0.58–7.01, *p* = 0.271, respectively) (Supplementary Table S2). However, after adjustment for age, sex, hypertension, diabetes mellitus, eGFR, and NT-proBNP, the association between log MPO and increased risk of MACEs and death at 6 months became nonsignificant (HR: 2.34, 95% CI: 0.98–5.61, *p* = 0.056; HR: 3.08, 95% CI: 0.94–10.06, *p* = 0.062, respectively) (Supplementary Table S2).

### Association Between TMAO Level and Clinical Outcomes

Clinical characteristics of the subjects stratified by median plasma TMAO levels are shown in Supplementary Table S3. Kaplan–Meier analyses suggest an increased risk for MACEs and death in patients with high plasma TMAO levels than those with low TMAO levels at 30 days (log-rank *p* = 0.004 and log-rank *p* = 0.002, respectively) and 6 months (log-rank *p* < 0.001 and log-rank *p* < 0.001, respectively) (Supplementary Figure S3). Cox regression analyses show that log TMAO predicted the risk of MACEs and death at 30 days (HR: 6.53, 95% CI: 2.13–19.98, *p* = 0.001; HR: 8.37, 95% CI: 2.29–30.67, *p* = 0.001, respectively) and 6 months (HR: 4.50, 95% CI: 1.78–11.40, *p* = 0.002; HR: 5.55, 95% CI: 1.81–17.02, *p* = 0.003, respectively) (Supplementary Table S2). However, after adjustment for age, sex, hypertension, diabetes mellitus, eGFR, and NT-proBNP, log TMAO was not found to be an independent predictor of MACEs and death at 30 days (HR: 3.37, 95% CI: 0.89–12.70, *p* = 0.073; HR: 3.34, 95% CI: 0.62–17.90, *p* = 0.160, respectively) and 6 months (HR: 2.19, 95% CI: 0.73–6.59, *p* = 0.161; HR: 1.67, 95% CI: 0.40–7.04, *p* = 0.486, respectively) (Supplementary Table S2).

### Association Between MPO Combined With TMAO and Clinical Outcomes

**FIGURE 1:**
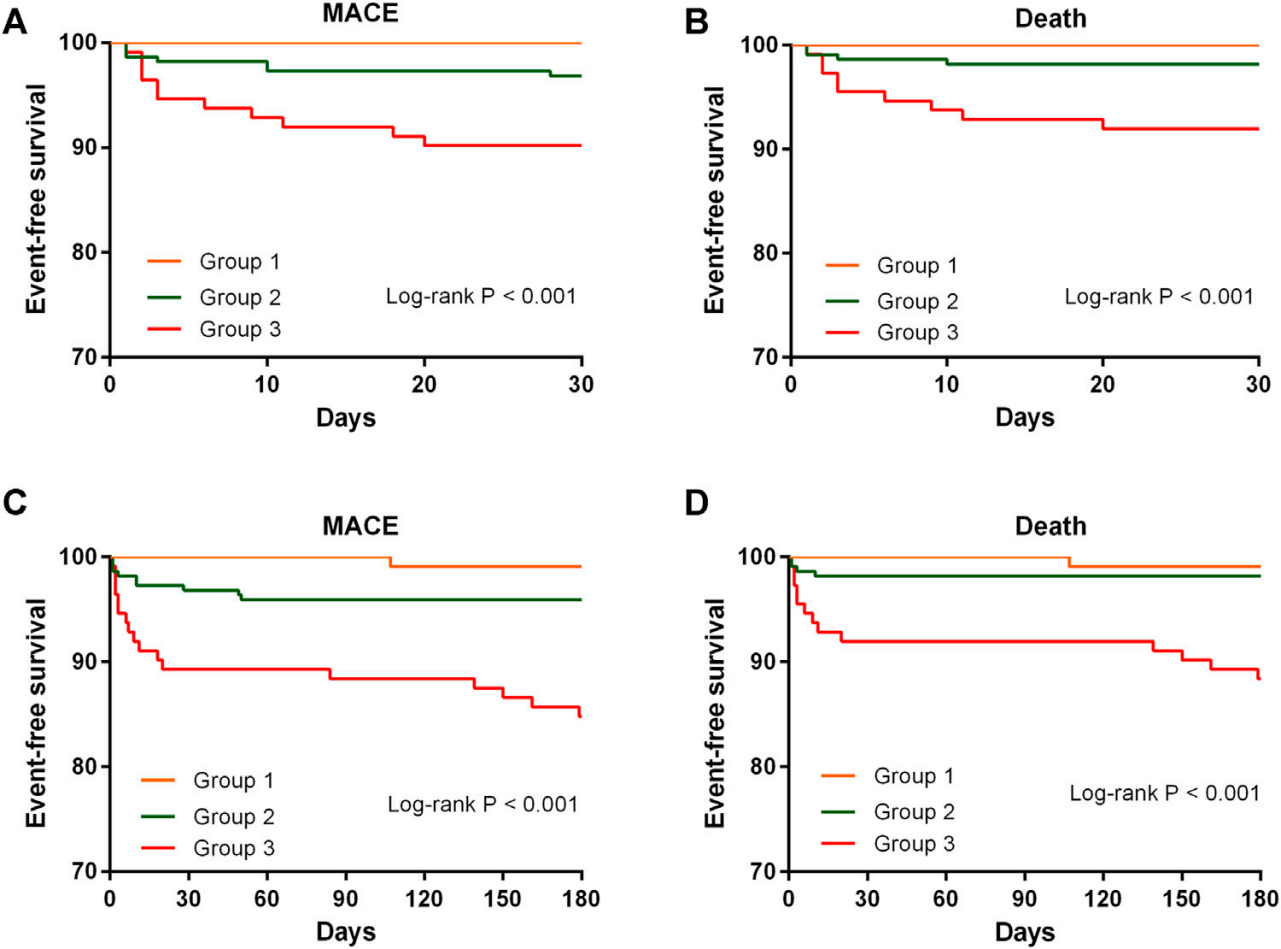
Kaplan-Meier survival curves of event-free survival stratified by the median level of MPO and TMAO for MACE and death at 30 days and 6 months. MACE, major adverse cardiovascular event; MPO, myeloperoxidase; TMAO, trimethylamine N-oxide.

As no previous study has investigated the ability of MPO combined with TMAO to predict clinical outcomes, we categorized patients into three groups based on the median level of plasma MPO and TMAO (Table 1). Group 1 had patients with plasma MPO and TMAO levels below median, group 2 had patients with either MPO or TMAO above median, group 3 had patients with MPO and TMAO above median. Kaplan–Meier analyses revealed a graded increased risk for MACEs and death from groups 1 to 3 at 30 days and 6 months (all log-rank *p* < 0.001) (Figure 1). Compared with subjects with low plasma levels of MPO and TMAO, patients with high plasma levels of MPO and TMAO demonstrated a significantly increased risk of MACEs (HR: 18.07, 95% CI: 2.40-135.78; *p* = 0.005) and death (HR: 13.51, 95% CI: 1.77-103.26; *p* = 0.012) at 6 months (Table 2). After adjustment for age, sex, hypertension, diabetes mellitus, eGFR, and NT-proBNP, high plasma levels of MPO and TMAO remained an independent predictor of MACEs at 6 months (HR: 11.88, 95% CI: 1.54–91.46, *p* = 0.017) (Table 2). However, high plasma levels of MPO and TMAO were not found to independently predict death at 6 months after adjustment (HR: 5.93, 95% CI: 0.73–46.42, *p* = 0.096) (Table 2). Notably, Cox regression analyses were not performed at 30 days because no patients underwent MACEs and death in group 1.

**TABLE 2:** Association between MPO combined with TMAO and major adverse cardiovascular event at 6 months.

	HR (95% CI) for MACEs		HR (95% CI) for death	
	Unadjusted	Adjusted	Unadjusted	Adjusted
Group 1	1.00	1.00	1.00	1.00
Group 2	4.62 (0.59–36.45)	4.14 (0.52–32.86)	2.03 (0.23–18.16)	1.61 (0.18–14.51)
Group 3	18.07 (2.40–135.78)^†^	11.88 (1.54–91.46)*	13.51 (1.77–103.26)*	5.83 (0.73–46.42)

Adjustments were made for age, gender, hypertension, diabetes mellitus, smoking, log-transformed eGFR and log-transformed NT-proBNP. CI, confidence interval; HR, hazard ratio; MACE, major adverse cardiovascular event; MPO, myeloperoxidase; TMAO, trimethylamine N-oxide; Group 1: both TMAO < median and MPO < median; Group 2: either TMAO > median or MPO > median; Group 3: both TMAO > median and MPO > median.

* p <0.05.

† p <0.01.

### Receiver-Operator Characteristic Curve Analysis and Reclassification Analysis

The AUC of MPO, TMAO, GRS, and the addition of MPO and TMAO to GRS was 0.642 (95% CI: 0.533–0.751, *p* = 0.013), 0.692 (95% CI: 0.595–0.790, *p* = 0.001), 0.736 (95% CI: 0.632–0.840, *p* < 0.001), and 0.760 (95% CI: 0.663–0.858, *p* < 0.001) for predicting MACEs, and 0.675 (95% CI: 0.553–0.797, *p* = 0.012), 0.724 (95% CI: 0.611–0.838, *p* = 0.001), 0.837 (95% CI: 0.746–0.929, *p* < 0.001), and 0.857 (95% CI: 0.770–0.944, *p* < 0.001) for predicting death at 6 months (Table 3). The AUC did not increase significantly when MPO and TMAO was added to GRS for predicting MACEs (*p* = 0.087) and death (*p* = 0.118) at 6 months (Table 3). Similar results were observed for predicting MACEs and death at 30 days (Table 3). However, addition of MPO and TMAO to GRS significantly improved the net reclassification of GRS alone in predicting MACE and death at 6 months with a NRI of 0.42 (*p* = 0.032) and 0.67 (*p* = 0.005) (Table 4). The net reclassification was also significantly improved when adding MPO and TMAO to GRS for predicting MACEs (NRI: 0.64, *p* = 0.022) and death (NRI: 0.49, *p* = 0.037) at 30 days (Table 4).

**TABLE 3:** Improvement for the addition of MPO and TMAO to GRS in predicting risk of cardiovascuar even assessed by area under the curve.

	GRS	GRS + TMAO	GRS + MPO	GRS + TMAO + MPO
6-month death				
AUC	0.837 (0.746-0.929)	0.842 (0.754—0.930)	0.852 (0.762-0.942)	0.857 (0.770-0.944)
P _difference_	—	0.419	0.187	0.118
6-month MACE				
AUC	0.736 (0.632—0.840)	0.743 (0.643-0.842)	0.753 (0.652-0.854)	0.760 (0.663-0.858)
P _difference_	—	0.319	0.150	0.087
1-month death				
AUC	0.816 (0.701—0.930)	0.824 (0.715-0.934)	0.818 (0.701-0.934)	0.827 (0.715-0.938)
P _difference_	—	0.454	0.551	0.328
1-month MACE				
AUC	0.723 (0.589—0.858)	0.747 (0.629-0.865)	0.724 (0.589-0.859)	0.747 (0.627-0.866)
P _difference_	—	0.203	0.458	0.175

AUC, area under the receiver operating characteristic curve; GRS, the global registry of acute coronary events (GRACE) risk score; MACE, major adverse cardiovascular event; MPO, myeloperoxidase; TMAO, trimethylamine N-oxide.

**TABLE 4:** Improvement for the addition of MPO and TMAO to GRS in predicting risk of cardiovascular event assessed by net reclassification index.

	GRS + MPO		GRS + TMAO		GRS + MPO + TMAO	
	NRI (95%CI)	*p*-value	NRI (95%CI)	*p*-value	NRI (95%CI)	*p*-value
6-month death	0.39 (−0.06–0.85)	0.092	0.47 (0.01–0.93)	0.050	0.67 (0.20–1.13)	0.005
6-month MACE	0.33 (−0.04–0.71)	0.080	0.35 (−0.03–0.73)	0.071	0.42 (0.04–0.80)	0.032
1-month death	0.17 (−0.27–0.62)	0.044	0.62 (0.15–1.08)	0.010	0.49 (0.03–0.96)	0.037
1-month MACE	0.28 (−0.26–0.81)	0.310	0.57 (0.02–1.12)	0.041	0.64 (0.09–1.19)	0.022

CI, confidence interval; GRS, the global registry of acute coronary events (GRACE) risk score; MACE, major adverse cardiovascular event; MPO, myeloperoxidase; NRI, net reclassification index; TMAO, trimethylamine N-oxide.

## Discussion

In this study, we demonstrate that plasma MPO and TMAO each is a univariate predictor of near-term clinical outcomes, including MACEs and death, in patients with STEMI. We further find that the combination of MPO and TMAO enhance the predictive value of MPO or TMAO alone. Moreover, the addition of MPO and TMAO to GRS can improve the ability of GRS alone in predicting near-term adverse events in STEMI patients.

MPO, a hemoprotein stored in azurophilic granules of leukocytes and released on neutrophil activation, generates numerous reactive oxidants and diffusible radical species that are capable of initiating lipid peroxidation (Rausch et al., 1978; Zhang et al., 2002a; Zhang et al., 2002b). A number of studies report that circulating MPO levels independently predict adverse cardiovascular events in patients with chest pain, acute coronary syndrome, or AMI (Baldus et al., 2003; Brennan et al., 2003; Khan et al., 2007; Mocatta et al., 2007; Morrow et al., 2008). However, some other studies observe no significant association between plasma MPO level on presentation and prognosis in patients with STEMI. (Kacprzak and Zielinska, 2016). In this study, we find that plasma MPO is able to predict MACEs and death at 6 months. However, the association became nonsignificant after adjustment for several traditional cardiovascular risk factors. It is worth noting that the *p* value for MACEs (*p* = 0.056) and death (*p* = 0.062) remained marginally significant, and this might be attributed to the small sample size and short follow-up. Mechanical studies demonstrate that MPO can induce endothelial cell apoptosis and tissue factor expression (Sugiyama et al., 2004), which are involved in thrombus formation and result in adverse cardiovascular events.

TMAO, a metabolite generated by the gut microbiota from dietary nutrients rich in choline, phosphatidylcholine, and L-carnitine, is shown to be atherogenic (Wang et al., 2011; Koeth et al., 2013). Numerous studies demonstrate an association between circulating TMAO levels and a poor prognosis in patients with either stable coronary artery disease or acute coronary syndrome (Tang et al., 2013; Wang et al., 2014b; Li et al., 2017; Suzuki et al., 2017). Our study extends previous findings by demonstrating a significant association between plasma TMAO level and increased risk of near-term adverse outcomes in patients with STEMI. Notably, the association became nonsignificant after adjustment for several risk factors. This finding is contradictory to the results of previous studies. The inconsistency in results might be attributed to the relatively small number of patients enrolled in the current study and low event rate, which indicates the need for a larger cohort and long-term follow-up. It is noteworthy that plasma TMAO levels varied enormously between studies, which might be attributed to geographical and ethnic factors, as the plasma TMAO levels in our study are similar to a previous study conducted by Liu et al. (2018), which also comprises Chinese ACS patients. The precise mechanism through which TMAO is involved in adverse clinical outcomes remains unclear; nevertheless, several studies provide some useful insights. An *in vivo* study demonstrates that TMAO promotes platelet hyperreactivity and thrombosis risk by enhancing stimulus-dependent release of Ca^2+^ from intracellular Ca^2+^ stores (Zhu et al., 2016). Numerous studies report an important role of platelet hyperreactivity in the incidence of thrombotic events (Trip et al., 1990; Marcucci et al., 2014). In addition, *in vitro* mechanistic studies indicate that TMAO can induce vascular inflammation and endothelial dysfunction through activating NLRP3 inflammasome and the mitogen-activated protein kinase/nuclear factor-kappa B pathway (Seldin et al., 2016; Boini et al., 2017; Chen et al., 2017), which also plays a critical role in thrombus formation.

Despite the advances in revascularization techniques, medical treatment, and risk factor modification, the prognosis of AMI remains poor (Vedanthan et al., 2014), indicating that more effective risk assessment is needed to improve the clinical outcome of AMI. The combination of biomarkers that reflect diverse pathophysiologic processes are appealing as an approach to provide complementary predictive value to enhance risk assessment and possibly target therapy more effectively (Sabatine et al., 2002; Morrow and Braunwald, 2003). Plasma MPO and TMAO concentrations are reported to be associated with the two main pathological mechanisms of AMI, plaque erosion and plaque rupture, respectively (Ferrante et al., 2010; Fu et al., 2016), and both of them could predict adverse events risk of patients with AMI (Baldus et al., 2003; Khan et al., 2007; Mocatta et al., 2007; Morrow et al., 2008; Wang et al., 2014b; Li et al., 2017; Suzuki et al., 2017). Therefore, simultaneous evaluation of the two biomarkers may provide complementary information in diagnosing the culprit plaque morphology and enable more accurate risk stratification and personalized treatment strategy. In this study, we find that combination of MPO and TMAO could enhance the prediction ability of near-term adverse outcomes in patients with STEMI when compared with MPO or TMAO alone.

GRS is an established powerful model of predicting prognosis for patients with ACS. However, it does not contain pathophysiologic variables, which could affect clinical outcomes of ACS patients. In this study, we investigate whether the addition of MPO and TMAO to GRS could improve the ability of GRS in predicting the prognosis of STEMI patients. Although the increments in AUC were not significant, the net reclassification analyses show that the combination of MPO and TMAO with GRS significantly improved the predictability of GRS alone for predicting near-term adverse events. It is not surprising that the increment in the AUC is not significant because ΔAUC provided by a new biomarker is overly conservative and depends on the performance of the underlying clinical model, which means that a powerful clinical model, such as GRS is difficult to improve (Chen et al., 2012). To deal with this anomaly, NRI is recommended to assess reclassification with novel biomarkers (Pencina et al., 2008). The significant improvements in net reclassification when adding MPO and TMAO to GRS may indicate a more accurate near-term risk stratification for patients with STEMI compared with GRS alone.

### Limitations

This study has several limitations. First, the predictive value of the combination of MPO and TMAO for long-term prognosis is not investigated because this is a contemporary study cohort. We aim to investigate this in future studies. Second, as this is a single center study, a multicenter validation cohort is needed to confirm our study results. Third, blood samples were collected at a single time point before emergency interventional procedures, and we did not have information about dietary history and prior antibiotic use for patients enrolled, which might have some impacts on plasma TMAO levels.

## Conclusion

High plasma levels of MPO and TMAO predict increased risk of near-term adverse clinical outcomes of patients with STEMI. The combination of MPO and TMAO could enhance the prediction ability of adverse events in STEMI patients when compared with MPO or TMAO alone. Moreover, the addition of MPO and TMAO could significantly improve the ability of GRS to predict near-term prognosis, suggesting a potential utility for the combination of MPO, TMAO, and GRS in improving risk stratification and clinical management of patients with AMI.

## Data Availability

The raw data supporting the conclusion of this article will be made available by the authors, without undue reservation.
