# Supplementary material for: Addition of Plasma Myeloperoxidase and Trimethylamine N-Oxide to the GRACE Score Improves Prediction of Near-Term Major Adverse Cardiovascular Events in Patients With ST-Segment Elevation Myocardial Infarction
Source: Front Pharmacol. 2021 Sep 28;12:632075. doi: 10.3389/fphar.2021.632075 (PMC8505536; doi:10.3389/fphar.2021.632075)
Supplement: Supplementary file 1 [file DataSheet1.docx]

**Supplemental materials**

**Supplemental Table 1.** Baseline characteristics categorized by median plasma MPO levels

|  | **All patients**  **(n=444)** | **MPO < median**  **(n=222)** | **MPO > median**  **(n=222)** | **P value** |
| --- | --- | --- | --- | --- |
| Age, years | 59.8±12.4 | 60.7±11.9 | 59.0±12.9 | 0.149 |
| Male, % | 81.1 | 82.0 | 80.2 | 0.628 |
| Medical history, % |  |  |  |  |
| Hypertension | 60.1 | 60.8 | 59.7 | 0.816 |
| Diabetes mellitus | 30.2 | 33.8 | 26.7 | 0.104 |
| Smoking | 65.5 | 63.5 | 67.4 | 0.387 |
| Laboratory tests |  |  |  |  |
| TC, mmol/L | 4.4±1.1 | 4.4±1.1 | 4.4±1.1 | 0.482 |
| LDL-C, mmol/L | 2.8±0.9 | 2.8±1.0 | 2.8±0.9 | 0.648 |
| HDL-C, mmol/L | 1.1±0.3 | 1.1±0.3 | 1.1±0.3 | 0.761 |
| Triglyceride, mmol/L | 1.4 (1.0-2.0) | 1.3 (1.0-1.9) | 1.4 (1.0-2.0) | 0.120 |
| eGFR, mL/min/1.73 m^2^ | 93.5 (75.8-108.9) | 93.8 (75.7-108.9) | 93.2 (77.1-108.7) | 0.262 |
| NT-proBNP, pg/mL | 167.0 (42.8-788.0) | 162.9 (39.9-592.2) | 167.0 (48.6-912.1) | 0.293 |
| MPO, ng/mL | 54.3 (34.5-109.7) | 34.5 (28.0-43.1) | 109.5 (73.9-193.9) | <0.001 |
| TMAO, μM | 2.40 (1.40-4.05) | 2.39 (1.54-4.07) | 2.40 (1.28-4.02) | 0.234 |
| Discharge medications, % |  |  |  |  |
| Aspirin | 97.3 | 97.7 | 96.8 | 0.558 |
| Statin | 96.4 | 96.8 | 95.9 | 0.611 |
| ACEI/ARB | 75.2 | 76.6 | 73.9 | 0.510 |
| Beta-blocker | 87.6 | 86.5 | 88.7 | 0.471 |
| GRACE score | 105 (84-123) | 107 (85-124) | 103 (83-122) | 0.203 |
| Endpoints, n (%) |  |  |  |  |
| 30 days |  |  |  |  |
| MACE | 18 (4.1) | 4 (1.8) | 14 (6.3) | 0.016 |
| Death | 13 (2.9) | 3 (1.4) | 10 (4.5) | 0.049 |
| 6 months |  |  |  |  |
| MACE | 27 (6.1) | 7 (3.2) | 20 (9.0) | 0.010 |
| Death | 18 (4.1) | 4 (1.8) | 14 (6.3) | 0.016 |

Continuous data are presented as mean ± standard deviation or median (interquartile range), categorical variables are presented as number (%).TC, total cholesterol; LDL-C, low density lipoprotein cholesterol; HDL-C, high density lipoprotein cholesterol; eGFR, estimated glomerular filtration rate; NT-proBNP, N-terminal pro-B-type natriuretic peptide; MPO, myeloperoxidase; ACEI/ARB, angiotensin-converting enzyme inhibitor/angiotensin receptor blocker; TMAO, trimethylamine N-oxide.

**Supplemental Table 2.** Cox regression analyses of MPO and TMAO in predicting adverse clinical outcomes at 30 days and 6 months.

|  | HR (95% CI) at 30 days | | | | HR (95% CI) at 6 Months | | | |
| --- | --- | --- | --- | --- | --- | --- | --- | --- |
|  | MACE | | Death | | MACE | | Death | |
|  | Unadjusted | Adjusted | Unadjusted | Adjusted | Unadjusted | Adjusted | Unadjusted | Adjusted |
| Log MPO | 1.72  (0.58-5.09) | 1.82  (0.59-5.81) | 2.02  (0.58-7.01) | 2.20  (0.50-9.60) | 2.55  (1.11-5.87)* | 2.34  (0.98-5.61) | 3.17  (1.18-8.55)* | 3.08  (0.94-10.06) |
| Log TMAO | 6.53  (2.13-19.98) † | 3.37  (0.89-12.70) | 8.37  (2.29-30.67) † | 3.34  (0.62-17.90) | 4.50  (1.78-11.40) † | 2.19  (0.73-6.59) | 5.55 (1.81-17.02) † | 1.67  (0.40-7.04) |

Adjustment were made for age, gender, hypertension, diabetes mellitus, smoking, log-transformed eGFR and log-transformed NT-proBNP. MPO, myeloperoxidase; TMAO, trimethylamine N-oxide; HR, hazard ratio; CI, confidence interval.

*P<0.05

†P<0.01

**Supplemental Table 3.** Baseline characteristics categorized by median plasma TMAO levels

|  | **All patients**  **(n=444)** | **TMAO < median**  **(n=221)** | **T MAO > median**  **(n=223)** | **P value** |
| --- | --- | --- | --- | --- |
| Age, years | 59.8±12.4 | 57.1±12.0 | 62.5±12.3 | <0.001 |
| Male, % | 81.1 | 81.0 | 81.2 | 0.963 |
| Medical history, % |  |  |  |  |
| Hypertension | 60.1 | 59.7 | 60.8 | 0.816 |
| Diabetes mellitus | 30.2 | 22.2 | 38.3 | <0.001 |
| Smoking | 65.5 | 67.9 | 63.1 | 0.287 |
| Laboratory tests |  |  |  |  |
| TC, mmol/L | 4.4±1.1 | 4.5±1.1 | 4.3±1.1 | 0.042 |
| LDL-C, mmol/L | 2.8±0.9 | 2.9±0.9 | 2.7±0.9 | 0.104 |
| HDL-C, mmol/L | 1.1±0.3 | 1.1±0.3 | 1.0±0.3 | 0.010 |
| Triglyceride, mmol/L | 1.4 (1.0-2.0) | 1.4 (1.1-1.9) | 1.4 (0.9-2.0) | 0.506 |
| eGFR, mL/min/1.73 m^2^ | 93.5 (75.8-108.9) | 97.8 (84.3-111.6) | 85.9 (66.9-104.1) | <0.001 |
| NT-proBNP, pg/mL | 167.0 (42.8-788.0) | 131.7 (37.3-548.0) | 213.7 (52.2-1115.9) | 0.010 |
| MPO, ng/mL | 54.3 (34.5-109.7) | 54.3 (35.1-116.7) | 54.4 (32.2-104.3) | 0.520 |
| TMAO, μM | 2.40 (1.40-4.05) | 1.40 (0.98-1.82) | 4.02 (3.22-6.80) | <0.001 |
| Discharge medications, % |  |  |  |  |
| Aspirin | 97.3 | 98.6 | 96.0 | 0.141 |
| Statin | 96.4 | 96.8 | 96.0 | 0.623 |
| ACEI/ARB | 75.2 | 75.6 | 74.9 | 0.869 |
| Beta-blocker | 87.6 | 89.1 | 86.1 | 0.331 |
| GRACE score | 105 (84-123) | 98 (79-114) | 113 (92-133) | P<0.001 |
| Endpoints, n (%) |  |  |  |  |
| 30 days |  |  |  |  |
| MACE | 18 (4.1) | 3 (1.4) | 15 (6.7) | 0.004 |
| Death | 13 (2.9) | 1 (0.5) | 12 (5.4) | 0.002 |
| 6 months |  |  |  |  |
| MACE | 27 (6.1) | 4 (1.8) | 23 (10.3) | <0.001 |
| Death | 18 (4.1) | 2 (0.9) | 16 (7.2) | 0.001 |

Continuous data are presented as mean ± standard deviation or median (interquartile range), categorical variables are presented as number (%).TC, total cholesterol; LDL-C, low density lipoprotein cholesterol; HDL-C, high density lipoprotein cholesterol; eGFR, estimated glomerular filtration rate; NT-proBNP, N-terminal pro-B-type natriuretic peptide; MPO, myeloperoxidase; ACEI/ARB, angiotensin-converting enzyme inhibitor/angiotensin receptor blocker; TMAO, trimethylamine N-oxide.





**Supplemental Figure 1.** Scatter plot showing the distribution of TMAO and MPO. MPO, myeloperoxidase; TMAO, trimethylamine N-oxide.





**Supplemental Figure 2.** Kaplan–Meier survival curves of event-free survival stratified by the median level of MPO for MACE and death at 30 days and 6 months. MACE, major adverse cardiovascular event.





**Supplemental Figure 3.** Kaplan–Meier survival curves of event-free survival stratified by the median level of TMAO for MACE and death at 30 days and 6 months. MACE, major adverse cardiovascular event.
